# Functional Foods Enriched With Bioactive Compounds: Therapeutic Potential and Technological Innovations

**DOI:** 10.1002/fsn3.71024

**Published:** 2025-10-07

**Authors:** Zargull Arshad, Shafa Shahid, Ammarah Hasnain, Esha Yaseen, Mehdi Rahimi

**Affiliations:** ^1^ Department of Biotechnology, Faculty of Biological Sciences Lahore University of Biological and Applied Sciences Lahore Pakistan; ^2^ Department of Biotechnology, Institute of Science and High Technology and Environmental Sciences Graduate University of Advanced Technology Kerman Iran; ^3^ Department of Medical Microbiology, College of Science Knowledge University Erbil Iraq

**Keywords:** bioactive compounds, biotechnological and AI‐driven approaches, carotenoids, gut microbiome modulation, omega 3‐fatty acids, polyphenols, probiotics and prebiotics

## Abstract

Functional foods have gained increasing attention for their dual role in providing essential nutrition and promoting health through the presence of bioactive compounds. These compounds, naturally found in a variety of plant and animal sources, include polyphenols, carotenoids, omega‐3 fatty acids, probiotics, prebiotics, alkaloids, and terpenoids. They exhibit a wide range of therapeutic effects, mediated through mechanisms such as antioxidant activity, anti‐inflammatory responses, modulation of gut microbiota, and enzyme inhibition. This review offers a comprehensive classification of these key bioactive compounds, detailing their natural origins with an emphasis on their mechanisms of action. Additionally, it explores their incorporation into diverse functional food matrices, including fortified beverages, dairy products, snack items, and dietary supplements. Modern biotechnological and AI‐driven approaches have revolutionized the precision, efficacy, and characterization of functional food products by enabling high‐throughput screening of bioactive compounds, predictive modeling for formulation, and large‐scale data mining to identify novel ingredient interactions and health correlations. Despite the growing popularity of functional foods, challenges persist in terms of the stability and bioavailability of bioactive compounds, regulatory hurdles, and consumer acceptance. Addressing these issues is critical to ensuring the efficacy and safety of functional food products. The review also highlights future perspectives in the field, emphasizing the need for innovative delivery systems and multidisciplinary research to enhance the bioavailability, functionality, and accessibility of these products. By highlighting the challenges and proposing possible solutions, this review serves as a foundational reference for bridging the gap among researchers, healthcare professionals, and stakeholders.

## Graphical abstract

Functional foods enriched with bioactive compounds such as polyphenols, carotenoids, omega‐3 fatty acids, and probiotics provide therapeutic benefits through antioxidant, anti‐inflammatory, and gut‐modulating mechanisms. Advances in biotechnology and AI have improved screening, formulation, and delivery of these compounds in diverse food matrices including dairy, beverages, and supplements. Despite challenges in stability, bioavailability, and regulation, innovative strategies and multidisciplinary research offer promising solutions to enhance efficacy, safety, and consumer acceptance of functional foods.

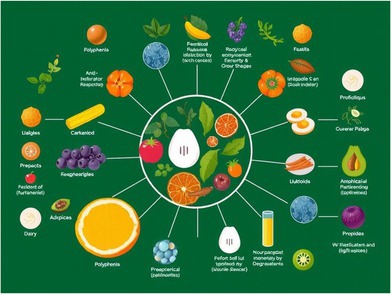

## 1 Introduction

**FIGURE 1:**
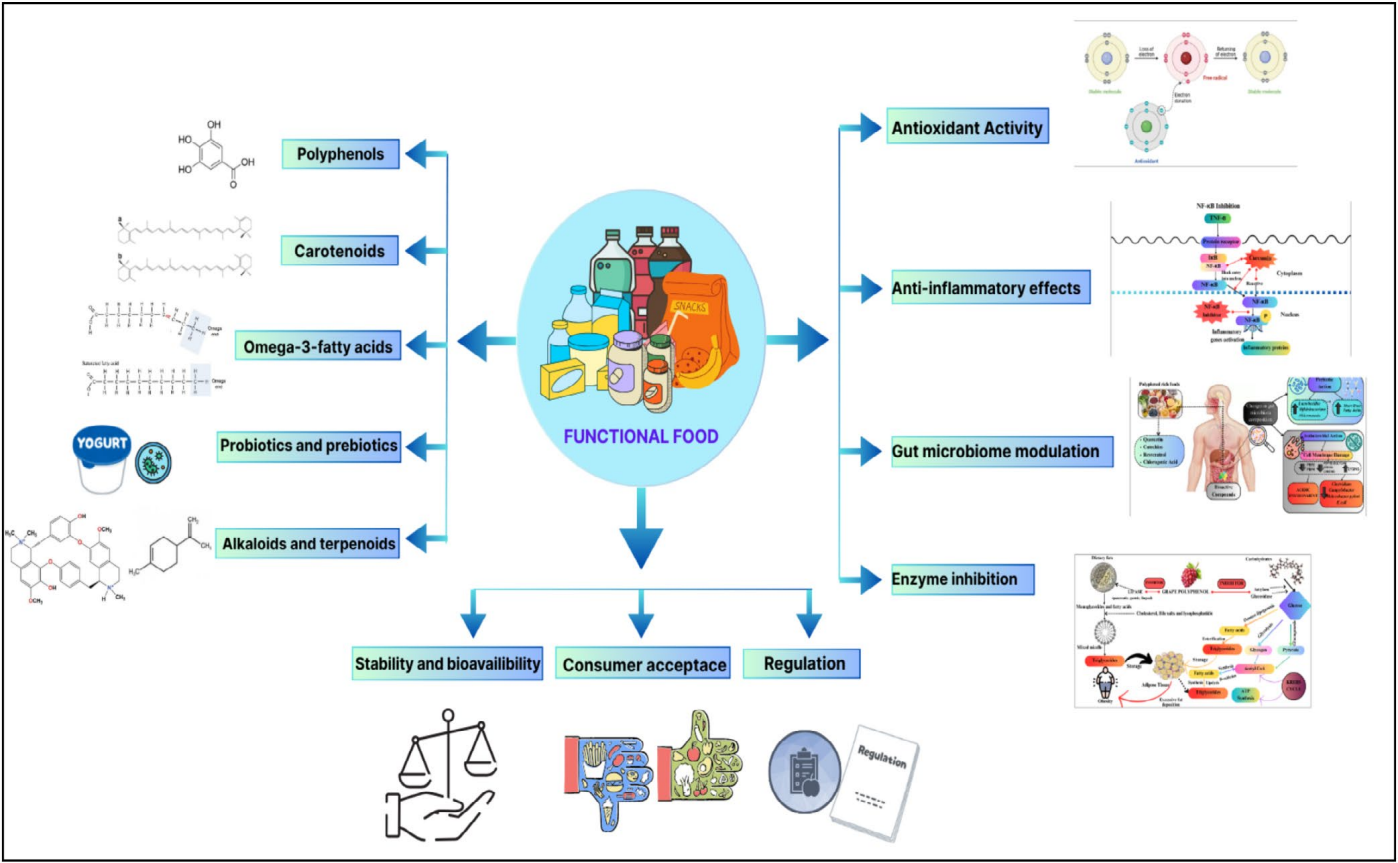
Functional food: types, activities, and future challenges.

Functional foods are dietary compounds that provide health benefits beyond basic nutrition due to the presence of crucial bioactive compounds such as polyphenols, carotenoids, omega‐3 fatty acids, alkaloids, isothiocyanates, plant stanols, sterols, flavonoids, polyols, soy protein, fatty acids, prebiotics, probiotics, phytoestrogens, as well as various minerals and vitamins (Shaikh 2022; Topolska et al. 2021). The concept of functional food originated in Japan during the 1980s, when government agencies began approving foods with verified health benefits (Arshad et al. 2021). The idea of functional foods has its roots in traditional dietary practices and has developed into scientifically formulated products designed to improve overall health and prevent diseases (Vignesh et al. 2024) (Figure 1).

In contrast to the conventional food that provides basic nutrition, functional foods are enriched with compounds that contribute to specific physiological effects (Table 1). Conventional foods provide essential nutrients required for survival while functional foods are enriched with bioactive ingredients that actively contribute to physiological well‐being (Hasler 2002; Victoria Obayomi et al. 2024).

**TABLE 1:** Comparison of conventional vs. functional foods based on formulation, regulation, and health benefits.

Feature	Conventional food	Functional food	References
Primary role	Provides essential nutrition	Offers health benefits beyond nutrition	Frumuzachi et al. (2025)
Formulation	Basic nutrients	Basic nutrients + bioactive compounds	Temple (2022)
Health claims	General	Specific	McClements and Xiao (2014)
Regulation	Standard food safety laws	Additional oversight for health‐related claims	Frumuzachi et al. (2025)
Examples	Rice, milk, bread	Probiotc yogurt, fortified cereals, omega‐3‐eggs	Tafese Awulachew (2024)

Key stages that involve in developing functional food are identification of beneficial compounds, their extraction from natural sources, and their incorporation into food matrices while ensuring stability, bioavailability, and efficacy (Rezagholizade‐shirvan et al. 2024). In addition to the key stages the ultimate step in developing functional food ensures that the food is palatable and acceptable to the consumers and this requires careful consideration of sensory properties, cost and convenience (Jones and Jew 2007; Vlaicu et al. 2023).

According to recent research, the bioactive compounds that are potentially found in food play a significant role in lowering the risk of chronic diseases, promoting gut health, reducing inflammation, boosting immune function, enhancing cognitive abilities, and assisting in weight management (Essa et al. 2023; Rivero‐Pino and Montserrat‐de la Paz 2024). The bioactive compounds, derived from plant‐based, marine, and microbial sources, have demonstrated great potential in addressing prevalent health concerns such as obesity, diabetes, cardiovascular diseases, and neurodegenerative disorders (Ud Din et al. 2023; Vignesh et al. 2024). The growing body of evidence supporting the health benefits of functional foods has led to their incorporation into dietary guidelines and health policies on a global scale (Xavier et al. 2024). The regulatory landscape for functional foods varies regionally, with some countries having established guidelines. Effectiveness relies on scientific validation, quality control, and labeling, requiring collaboration between food scientists, nutritionists, and regulatory agencies (Martirosyan and Alvarado 2023).

Recent meta‐analytic evidence indicates that polyphenols can significantly improve muscle mass in sarcopenic individuals, highlighting their therapeutic potential (Medoro et al. 2024). Omega‐3 fatty acid supplementation (0.8–1.2 g/day) significantly reduces the risk of major cardiovascular events, heart attacks, and cardiovascular death, especially in patients with coronary heart disease according to the meta‐analysis by Shen et al. (2022). Probiotic efficacy has been evaluated through meta‐analyses across conditions like irritable bowel syndrome (IBS), allergic rhinitis, and pediatric atopic dermatitis, offering stronger evidence on their therapeutic and preventive benefits (Farahmandi et al. 2022; Lee et al. 2008).

The development of functional foods encounters several challenges, such as consumer perception, affordability, and the sustainability of sourcing bioactive ingredients. Some consumers are still doubtful about the health benefits associated with functional foods, highlighting the need for public education and clear scientific communication (Baker et al. 2022). Furthermore, it is important to ensure the sustainable production of bioactive compounds while keeping costs manageable to make functional foods accessible to a wide range of populations (Yuan et al. 2024).

In this review, crucial bioactive compounds, their natural origins, and their contribution to the creation of functional foods have been studied. By comprehending the characteristics and mechanisms of these bioactive compounds, researchers and food technologists can develop innovative functional foods that promote health and well‐being. Ongoing progress in food technology and nutrigenomics offers promising opportunities to enhance the effectiveness of functional foods, leading to a healthier future.

## 2 Key Bioactive Compounds and Their Sources

### 2.1 Polyphenols as Potent Antioxidants and Disease Modulators

Polyphenols are one of the most prevalent classes of bioactive metabolites in plants, which are important for the human body through their impactful antioxidant, anti‐inflammatory, and antimicrobial activities. Such secondary metabolites are found in a wide range of dietary sources that include fruits, such as berries, apples, and grapes; vegetables, such as spinach, onions, and kale; tea and coffee; and whole grains (Table 1) (Pandey and Rizvi 2009).

Recent studies highlight the role of nanoencapsulation in enhancing the bioavailability and therapeutic effectiveness of polyphenols. Encapsulation techniques improve stability, protect polyphenols from degradation, and enhance absorption in the body, making them more effective in disease prevention and treatment (Ali Redha et al. 2024; Pugazhendhi et al. 2025).

### 2.2 Carotenoids as Pigments With Nutritional and Therapeutic Potential

Carotenoids are lipophilic pigments widely distributed in nature, known for their dual significance in human health. They can act as both provitamin A carotenoids and preformed vitamin A, playing essential roles in human nutrition and disease prevention. Provitamin carotenoids are found in plant‐based sources like fruits and vegetables such as carrots, tomatoes, bell peppers, and leafy greens (Table 2) (Dumay and Morançais 2016). They contribute to the essential physiological functions including vision, immune response, and cellular growth (Huang et al. 2022). On the contrary, preformed vitamin A is found in foods from animal sources, including dairy products, eggs, fish, and organ meats. They contribute to potential pharmacological properties, particularly antioxidant, anti‐inflammatory, and anticancer activities, although these effects are still under investigation and not yet fully established for clinical trials (NIH 2025).

**TABLE 2:** Overview of bioactive compounds, their sources, key health benefits, daily intake threshold vs. pharmacological doses (mg/day).

Bioactive compounds	Examples/key functions	Major food sources	Key health benefits	Daily intake threshold (mg/day)	Pharmacological doses (mg/day)	References
Polyphenols						
Flavonoids	Quercetin, catechins, anthocyanins, kaempferol	Berries, apples, onions, green tea, cocoa, citrus fruits	Cardiovascular protection, anti‐inflammatory effects, antioxidant properties, improved blood circulation	300–600	500–1000	Ullah et al. (2020)
Phenolic Acids	Caffeic acid, ferulic acid, gallic acid	Coffee, whole grains, berries, spices (e.g., cinnamon), olive oil	Neuroprotection, antioxidant activity, reduced inflammation, skin health benefits	200–500	100–250	Mihaylova et al. (2024)
Lignans	Secoisolarici‐ ‐resinol, matairesinol	Flaxseeds, sesame seeds, whole grains, legumes	Hormone regulation, cancer prevention, improved gut microbiota, cardiovascular benefits	~1	50–600	Gass and Khan (2013)
Stilbens	Resveratrol, pterostilbene	Red wine, grapes, peanuts, blueberries	Anti‐aging effects, cardiovascular protection, anticancer properties, cognitive health improvement	~1	150–500	Kaur et al. (2024)
Carotenoids						
Beta‐carotene	Provitamin A compound	Carrots, sweet potatoes, spinach, mangoes, pumpkin	Supports immune function, enhances vision, promotes skin health	2–7	15–30	Sharma et al. (2024)
Luten	Eye health, blue light filtration	Kale, spinach, broccoli, corn, egg yolk	Protects against age‐related macular degeneration (AMD), reduces eye strain	1–3 mg/day	10–20 mg/day	Mrowicka et al. (2022)
Zeaxanthin	Ocular protection, antioxidant properties	Orange bell peppers, corn, goji berries, eggs	Reduces the risk of cataracts and AMD, improves visual function	0.3–1.0 mg/day	2–10 mg/day	Mrowicka et al. (2022)
Alpha‐linolenic acid (ALA)	Precursor to EPA & DHA, supports metabolic functions	Flaxseeds, chia seeds, walnuts, soybeans	Cardiovascular protection, anti‐inflammatory effects	1.1 g/day (women) 1.6 g/day (men) as per US Institute of Medicine	2–5 g/day	Rajaram (2014)
Eicosapentaenoic acid (EPA)	Reduces systemic inflammation, regulates blood clotting	Fatty fish (salmon, mackerel, sardines), krill oil, algae‐based supplements	Lowers triglyceride levels, reduces risk of hypertension & atherosclerosis	~100–250 mg/day	500–4000 mg/day	Nassar et al. (2023)
Docosahexaenoic acid (DHA)	Essential for brain & retinal development, supports cognitive function	Fatty fish, fish oil, algae oil	Protects against neurodegenerative disorders (e.g., Alzheimer's), enhances memory & learning	~100–200 mg/day	300–2000 mg/day	Ahmad et al. (2024)
Alkaloids and terpenoids						
Alkaloids	Caffeine, theobromine, morphine, nicotine, berberine	Tea, coffee, cocoa, opium poppy, berberis, tobacco	Antimicrobial, analgesic, neuroprotective, anti‐inflammatory, CNS stimulation	Usually low or trace	Moderate to high (1–1500 mg/day)	Behl et al. (2022)
Terpenoids	Limonene, curcumin, menthol, linalool, artemisinin	Citrus peels, turmeric, rosemary, mint, lavender, artemisia	Antioxidant, anti‐inflammatory, antimicrobial, anticancer, neuroprotective	Low via diet (< 1–10 mg/day)	100–1000+ mg/day	Sharma et al. (2017)

Modification of carotenoid content in staple crops through genetic modification is one of the biofortification strategies that have been explored to overcome vitamin A deficiency in susceptible populations (Naik et al. 2024). For instance, Golden Rice, a genetically modified crop developed to combat vitamin A deficiency (VAD) by producing provitamin A (β‐carotene) in the edible endosperm through the insertion of psy (from maize) and crtI (from Pantoea ananatis) genes. The β‐carotene in Golden Rice is efficiently converted to vitamin A in humans. It has been declared safe for human consumption by major regulatory agencies including US FDA and Health Canada based on assessments from the International Rice Research Institute (IRRI) (IRRI 2025; ISAAA 2018). The World Health Organization (WHO) considers biofortification, including genetic modification, a promising and sustainable strategy to combat micronutrient deficiencies in populations with limited dietary diversity. However, WHO highlights the need for rigorous safety assessments, adherence to Codex Alimentarius standards, long‐term monitoring, and community acceptance before issuing formal recommendations (WHO 2023).

### 2.3 Omega‐3 Fatty Acids as Essential Lipids for Brain and Heart Health

**FIGURE 2:**
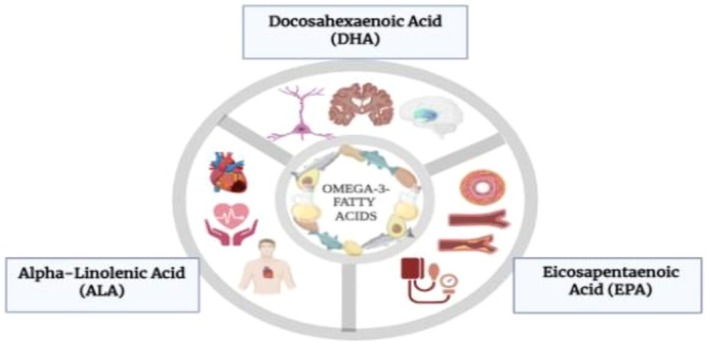
Sources and health benefits of omega‐3 fatty acids.

Omega‐3 fatty acids, a class of essential polyunsaturated fatty acids (PUFAs), are well recognized for their profound impact on cardiovascular, cognitive, and metabolic health (Figure 2). These bioactive lipids are predominantly found in fatty fish such as salmon, mackerel, and sardines, as well as in plant‐based sources like flaxseeds, chia seeds, and walnuts (Table 3).

**TABLE 3:** Overview of probiotics, prebiotics, and synbiotics, their sources, and key health benefits.

Category	Key microorganisms/components	Major food sources	Health benefits	References
Probiotics	*Lactobacillus*, *Bifidobacterium*, *Saccharomyces boulardii*	Yogurt, kefir, sauerkraut, kimchi, miso, kombucha	Improves digestion, enhances immune response, reduces risk of IBS and IBD, restores gut flora after antibiotic use	Kothari et al. (2019)
Prebiotics	Inulin, fructooligosaccharides (FOS), galactooligosaccharides (GOS), resistant starch	Garlic, onions, bananas, whole grains, asparagus, chicory root	Promotes growth of probiotics, enhances gut microbiota diversity, supports digestive health, reduces inflammation	Victoria Obayomi et al. (2024)
Synbiotics	Probiotic strains + Prebiotic fibers	Fermented dairy with added prebiotics, functional foods, dietary supplements	Improves microbiota composition, enhances nutrient absorption, potential role in mental health via gut‐brain axis	Markowiak and Śliżewska (2017)

### 2.4 Alkaloids and Terpenoids as Bioactive Compounds With Medicinal Properties

Alkaloids and terpenoids are two significant classes of bioactive compounds found in plants, each with diverse pharmacological properties. Alkaloids are nitrogen‐containing secondary metabolites that exhibit antimicrobial, analgesic, neuroprotective, and anti‐inflammatory activities. These compounds are prevalent in various medicinal plants, as well as in widely consumed beverages such as tea, coffee, and cocoa (Heinrich et al. 2021). Notable alkaloids include caffeine, theobromine, and morphine, each of which exerts distinct physiological effects. Caffeine, for instance, acts as a central nervous system stimulant, enhancing alertness and cognitive function, while morphine is a potent analgesic used in pain management (Evans et al. 2025) (Table 2).

Terpenoids, also known as isoprenoids, represent the largest class of plant‐derived natural compounds with extensive medicinal and nutritional applications. These lipophilic compounds contribute to the aroma, flavor, and pigmentation of many plants and are commonly found in essential oils, citrus fruits, and herbs such as rosemary and mint (Ludwiczuk et al. 2017). Terpenoids possess antioxidant, anti‐inflammatory, antimicrobial, and anticancer properties, making them valuable in pharmaceutical and nutraceutical industries (Siddiqui et al. 2024). Compounds such as limonene, found in citrus peels, have been studied for their potential role in cancer prevention, while curcumin, a bioactive terpenoid from turmeric, exhibits strong anti‐inflammatory and neuroprotective effects. With the rising interest in plant‐based medicines, the extraction, synthesis, and bioavailability enhancement of alkaloids and terpenoids remain key research areas for developing novel therapeutic agents (Koolaji et al. 2020) (Table 2).

### 2.5 Probiotics, Prebiotics, and Their Role in Gut Microbiota

The gut microbiome plays a pivotal role in human health, and probiotics and prebiotics are essential modulators of microbial diversity and function (Table 3).

Recent research highlights the gut‐brain axis as a key area of interest, suggesting that probiotics and prebiotics may influence mental health by modulating neurotransmitter production and reducing inflammation.

## 3 Mechanism of Action

### 3.1 Antioxidant Activity

**FIGURE 3:**
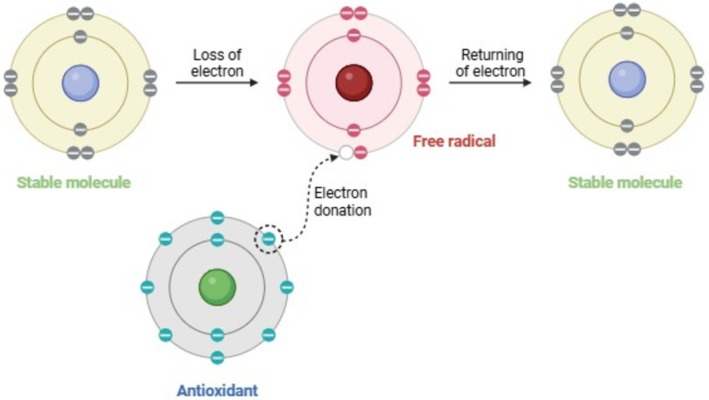
Principal mode of action of antioxidants.

Antioxidant activity is an ability of a molecule to prevent the oxidation of other useful molecules in the body, which results in the generation of harmful free radicals. Antioxidants are capable of donating electrons to free radicals, thus neutralizing these radicals and averting them from causing cellular and tissue damage (Figure 3) (Lu et al. 2021; Munteanu and Apetrei 2021). In functional foods, antioxidants are predominately phenolic in nature, such as flavonoids, polyphenols, and omega‐3 fatty acids, which chelate pro‐oxidant ionic metals and scavenge reactive oxidative species (ROS), thus reducing inflammation and oxidative stress (Abeyrathne et al. 2022; Uro‐Chukwu et al. 2025).

#### 3.1.1 Role of Dietary Antioxidants in Different Body Mechanisms

**FIGURE 4:**
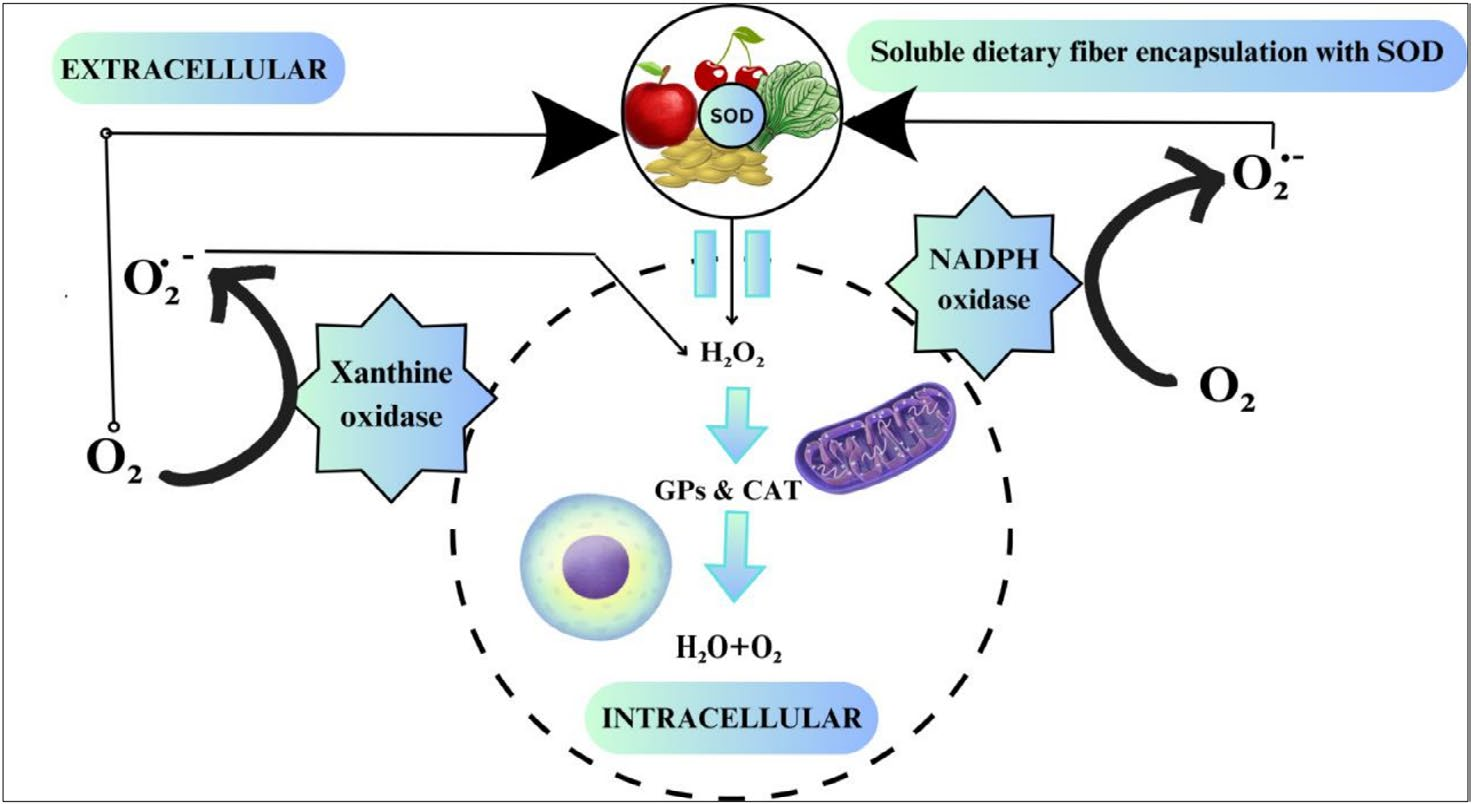
Dismutation of superoxide radicals into hydrogen peroxide by superoxide dismutase encapsulated in dietary fibers. https://www.canva.com/design/DAGjAUUKI40/WR2xVIX22XTqa6P0mVlX‐g/edit?utm_content=DAGjAUUKI40&utm_campaign=designshare&utm_medium=link2&utm_source=sharebutton.

The inhibition of oxidative stress is achieved by some common dietary antioxidants such as vitamin C and E, carotenoids, and polyphenols that donate electrons to stabilize free radicals, including peroxyl radicals (ROO·), superoxide anions ($O_{2}^{-}$) and hydroxyl radicals (OH·) (Hossain et al. 2022; Vignesh et al. 2024). Tomato‐based products that are enriched with carotenoid lycopene lower cardiovascular disease risk by reducing oxidative stress. Dietary fibers modulate enzymatic defense systems endogenously, such as superoxide dismutase (SOD), which catalyzes dismutation of superoxide radicals into hydrogen peroxide, which is broken down into oxygen and water (Figure 4) (Eddaikra and Eddaikra 2021; Islam et al. 2022).

Although antioxidants play a crucial role in reducing excessive reactive oxygen species (ROS) and preventing oxidative damage, it is important to recognize that moderate ROS levels are physiologically essential. ROS act as signaling molecules in pathways regulating cell proliferation, differentiation, immune responses, and stress adaptation. Complete elimination of ROS can disrupt these redox‐sensitive signaling cascades, impair adaptive responses, and compromise cellular homeostasis. Therefore, the aim of antioxidant‐based interventions should be to maintain redox balance, preserving beneficial ROS‐mediated functions while preventing harmful oxidative stress (Hasanuzzaman et al. 2020; Poljsak et al. 2013).

### 3.2 Anti‐Inflammatory Effects

Inflammation is a complicated physiological response that arises when the human body reacts to different adverse stimuli, such as toxic agents, cellular injury, and pathogenic microbes. Chronic inflammation significantly leads to cancer, diabetes, cardiovascular disease, and neurodegenerative diseases (Elbandy 2022; Ma et al. 2025). Functional foods have bioactive compounds that exert anti‐inflammatory benefits through several molecular mechanisms (Martirosyan et al. 2022; Mondal et al. 2021).

#### 3.2.1 Pro‐Inflammatory Cytokines Inhibition

Pro‐inflammatory cytokines such as interleukin‐1β (IL‐1β), interleukin‐6 (IL‐6), and tumor necrosis factor‐alpha (TNF‐α) play an important role in the inflammatory response, which is being suppressed by functional food bioactives (Coutinho‐Wolino et al. 2022). Different bioactive compounds are known for their anti‐inflammatory responses in various reports. According to previous studies on *Curcuma longa* (common name: turmeric), its bioactive compound curcumin, also known as diferuloylmethane, possesses anti‐inflammatory properties. The study reveals that 10–20 μM of curcumin concentrations significantly reduce IL‐6 and TNF‐α secretion in lipopolysaccharides (LPS)‐stimulated macrophages by inhibiting NF‐κB pathway and toll‐like receptors (TLR4) activation (Guimarães et al. 2013).

Moreover, omega‐3 fatty acids are also known to suppress inflammatory responses in living cells. Meta‐analytic data reported that daily intake of 30 mg of omega‐3 fatty acids for 12 weeks significantly reduced IL‐6 levels by 1.87 pg/mL and TNF‐α by 2.11 pg/mL in a clinical trial on mice models (Djuricic and Calder 2021; Kavyani et al. 2022; Roşian et al. 2025). In another clinical study on humans, omega‐3 fatty acid supplementation (2–4 g/day EPA and DHA) led to a 24%–36% reduction in serum IL‐6 and TNF‐α in patients with rheumatoid arthritis and cardiovascular disease (Table 4) (Banaszak et al. 2024; Bodur et al. 2025).

**TABLE 4:** Mechanism of action of various functional food bioactives in the inhibition of pro‐inflammatory cytokines.

Bioactive compound	Food sources	Mechanism of action	References
Curcumin	Turmeric	Inhibits ofNLRP3 and NF‐κBInflammasome (10–20 μM)	Barakat et al. (2023), Guimarães et al. (2013)
Quercetin	Onions, garlic	Inhibits NF‐κB and NLRP3 inflammasome activation (5‐50 μM)	Chojnacka and Lewandowska (2023), Djuricic and Calder (2021)
Resveratrol	Berries, grapes	Activation of SIRT1 for inhibiting cytokine expression	Zhang et al. (2022), Banaszak et al. (2024)
Omega‐3 fatty acids	Fatty fish	Inhibition of NF‐κB signaling by activation of PPAR‐γ (2–4 g/day)	Sharma et al. (2024), Cui et al. (2019)
Flavonoids	Fruits and vegetables	Modulation of NF‐κB, AP‐1, PPAR, Nrf2, and MAPK pathways	Behl et al. (2021)
Terpenes	Mushrooms	Inhibition of TLR4 activation (50–100 μg/mL)	Spano et al. (2022), Fakhri et al. (2022)

#### 3.2.2 NF‐κBsignaling Pathway Modulation

**FIGURE 5:**
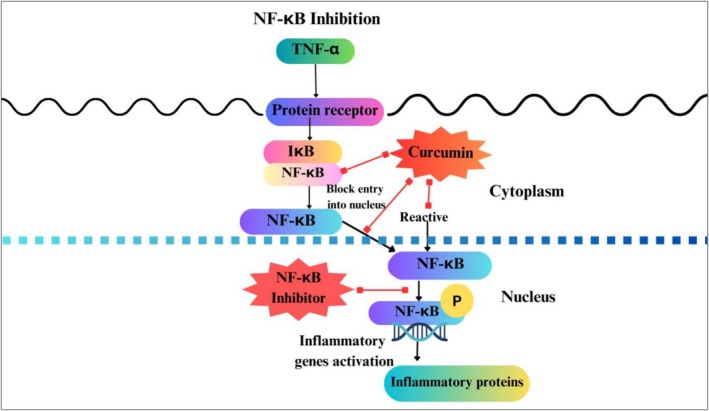
Inhibition of NF‐κBby curcumin. https://www.canva.com/design/DAGjFRImsNo/qR00qEtcxaAihr‐QyFuf4w/edit?utm_content=DAGjFRImsNo&utm_campaign=designshare&utm_medium=link2&utm_source=sharebutton.

NF‐κB pathway plays an important role in regulating inflammation, with its sustained activation associated with various ailments like neuroinflammation, cancer, and cardiovascular disease (Kannan et al. 2025). According to a recent study, 10 μΜ of curcumin was found to inhibit IκB kinase in murine macrophage cell lines (RAW264.7), thus preventing phosphorylation and degradation of IκB, hence blocking nuclear translocation of NF‐κB (Figure 5). Similarly, 25 μM of resveratrol inhibited NF‐κB activation in LPS‐stimulated THP‐1 human monocytes, leading to a significant decrease in TNF‐α by 40% (Fakhri et al. 2022; Patel et al. 2020). A polyphenolic compound, resveratrol that is found in grapes and red wine, averts activation of NF‐κb in monocytes, which results in reduced secretion of IL‐1β and TNF‐α (Chojnacka and Lewandowska 2023).

A well‐controlled study in mice fed a high‐fat diet (HFD) with resveratrol supplementation (30 mg/kg/day) demonstrated significant metabolic and anti‐inflammatory benefits, including decreased hepatic mRNA expression of NF‐κB, IL‐6, TNF‐α, and IL‐1β, increased SIRT1 expression, improved lipid metabolism, and reduced markers of NAFLD (e.g., liver triglycerides and steatosis) (Andrade et al. 2014; Yasmin et al. 2025; Zhou et al. 2019).

#### 3.2.3 MAPK Pathway Regulation

Mitogen‐activated protein kinase (MAPK) pathway is vital for cytokine synthesis by transmitting inflammatory signals (Ganguly et al. 2023). According to research, epigallocatechin gallate (EGCG) from green tea (25–50 μM) inhibits phosphorylation of p38 MAPK in macrophages, resulting in reduced IL‐1β and TNF‐α levels (Behl et al. 2021). Quercitin (30 μM) present in onions and apples inhibits the activation of JNK signaling in LPS‐stimulated RAW264.7 macrophages, contributing to decreased iNOS and COX‐2 expression (Cui et al. 2019; Sahu and Rawal 2024).

### 3.3 Gut Microbiome Modulation

Functional nutrients can modulate the gut microbiome by selectively promoting the growth of beneficial microbes, thereby influencing metabolic homeostasis. These nutrients play an important role in preventing gut microbiome imbalance by inhibiting pathogenic microbes and promoting the proliferation of bioactive microbial metabolites (Dahiya and Nigam 2022; Green et al. 2020). Prebiotics, including resistant starch, fructans, and galactooligosaccharides, act as substrates for beneficial gut bacteria such as *Lactobacillus* and *Bifidobacterium*. These bacteria produce short‐chain fatty acids (SCFA) such as butyrate, acetate, and propionate that maintain intestinal barriers (Zhang et al. 2023). Dietary fiber fermentation lowers oxidative stress and inflammation by strengthening the intestinal barrier and enhancing the integrity of tight junctions (Gao et al. 2022).

#### 3.3.1 Role of Dietary Polyphenols in Balancing Gut Microbiota

**FIGURE 6:**
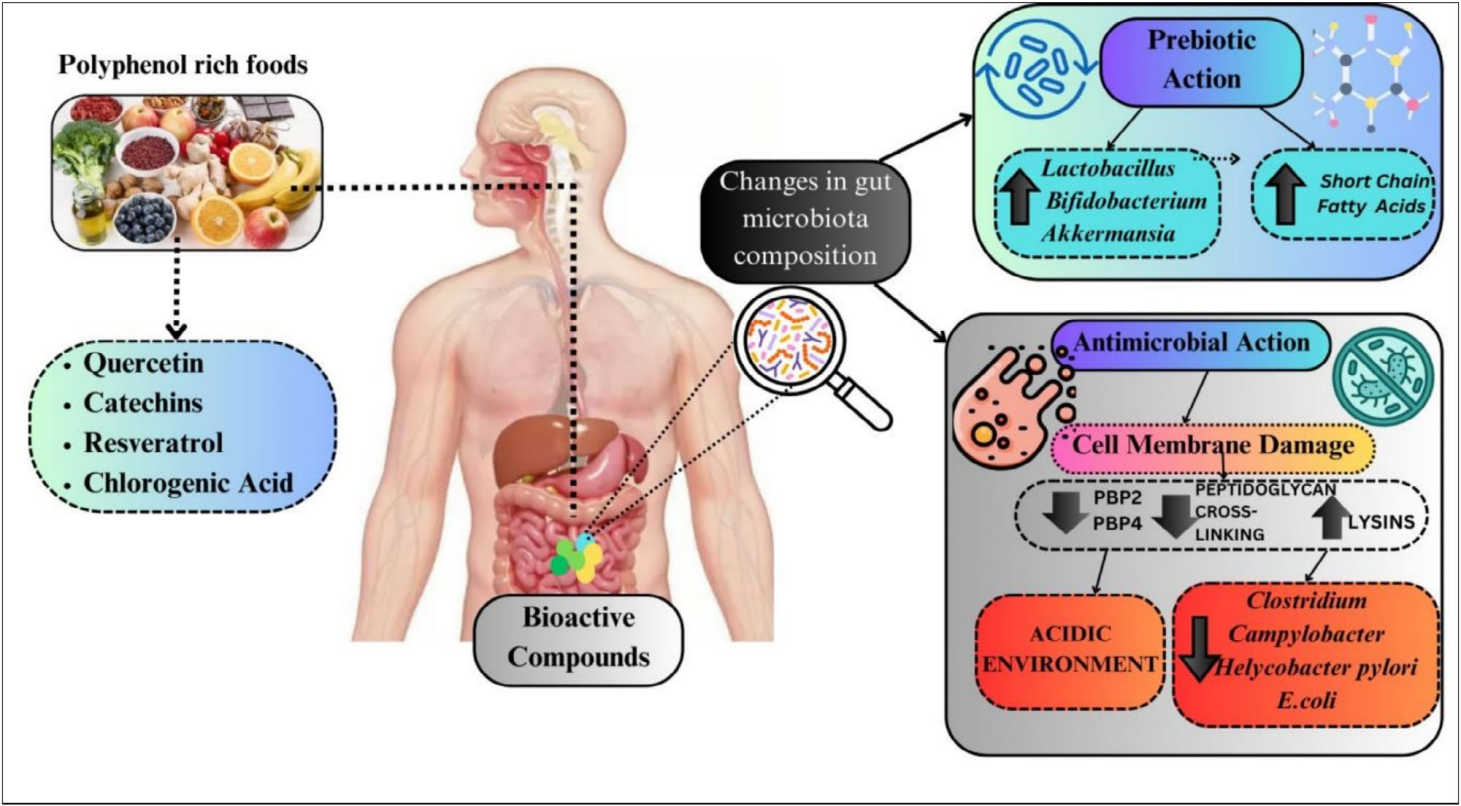
Impact of dietary polyphenols on gut microbiome. https://www.canva.com/design/DAGjG9xB8F4/0e6SeBhsRr3rbnbZKMw39g/edit?utm_content=DAGjG9xB8F4&utm_campaign=designshare&utm_medium=link2&utm_source=sharebutton.

Polyphenol‐rich foods such as blueberries, cranberries, tea, and cocoa enhance the proliferation of *Akkermansia muciniphila, Lactobacillus* sp., and *Bifidobacterium bifidum as* a prebiotic effect while suppressing obesity‐associated microorganisms and enhancing short‐chain fatty acid levels (Liu et al. 2020). They also play a significant role in suppressing harmful bacteria by inhibiting PBP2 and PBP4, which lead to a reduction in peptidoglycan cross‐linking and an increase in lysine, as well as the creation of an acidic environment in the gut by the deletion of H + ‐ATPase5, proton donation, and proton pump impairment (Figure 6). Flavonoids from citrus fruits and polyphenols from green tea have been shown to modulate bile acid metabolism and improve cholesterol homeostasis. In addition, omega‐3 fatty acids lead to bile acid conversion and gut microbial diversity, thus promoting inflammation‐free microbial diversity (Wang et al. 2024).

### 3.4 Enzyme Inhibition

Enzymes are essential for human survival; any malfunction in them can cause overexpression or underexpression of enzymes necessary for normal functioning. This can be stopped by the formation of an enzyme‐inhibitor complex that inhibits aberrant enzyme functioning (Phull et al. 2022; Saleem et al. 2024). Functional food phytochemical constituents regulate crucial enzymes, thus lowering the risk of diseases and promoting improved health. A number of functional foods contain key bioactive components that exert therapeutic effects by obstructing enzymes involved in carbohydrate metabolism, digestion of lipids, oxidative stress, and inflammatory pathways (Hou et al. 2024).

#### 3.4.1 Pancreatic Lipase Inhibition

**FIGURE 7:**
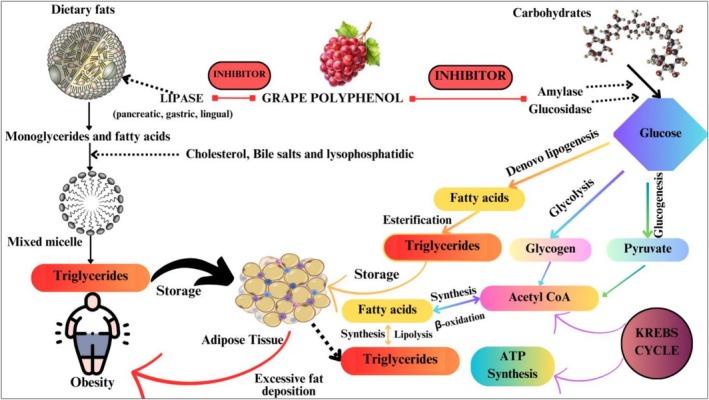
Inhibitory potential of pancreatic lipase by grape polyphenols. https://www.canva.com/design/DAGjLT3ibvs/AsxAmVlj7ADpCuFB5rULCg/edit?utm_content=DAGjLT3ibvs&utm_campaign=designshare&utm_medium=link2&utm_source=sharebutton.

Pancreatic lipase plays a crucial role in managing weight and the breakdown of dietary fats (Lim et al. 2022). Control of obesity and dyslipidemia can be managed via pancreatic lipase inhibition by functional bioactives, which is an effective approach. Grapes contain proanthocyanidins that bind to the active site of this enzyme, limiting its ability to break down into free fatty acids (Figure 7). Likewise, saponins in fenugreek and different legumes form insoluble complexes by interacting with dietary lipids, thereby restraining the bioavailability of fats (Jamai et al. 2024).

#### 3.4.2 Inhibition of Butyryl Cholinesterase (BChE) and Acetyl Cholinesterase (AChE)

Inhibition of butyryl cholinesterase and acetyl cholinesterase, enzymes linked to Alzheimer's disease pathology, is derived from functional food bioactives. According to research, about 12 active ingredients from *Ganoderma amboinense*, such as lucidenic acid F and ganoderic acid, exhibit AChE inhibition for Alzheimer's management. Enzymatic hydrolysates present in yellow field pea proteins, particularly those treated with flavourzyme, pepsin, and alcalase, resulted in inhibition of AChE activity by 20%–30% (Hou et al. 2024). Similarly, extracts of *Limonium spathulatum* showed substantial inhibitory action against BChE, with IC_50_ values of about as low as 0.03 mg/mL (Grzelczyk et al. 2023). However, despite promising in vitro enzyme inhibition, it is important to note that many phytochemicals face limited permeability across the blood–brain barrier, which can restrict their therapeutic efficacy in neurological disorders like Alzheimer's disease (Paramanick et al. 2022).

#### 3.4.3 Inhibition of Carbohydrate Digesting Enzymes

α‐Amylase and α‐glucosidase are carbohydrate‐digesting enzymes that are mandatory for the digestion and absorption of nutrients. However, their overexpression may lead to several metabolic issues (Vyas et al. 2024). Their inhibition is a strategy for controlling postprandial hyperglycemia, which is the major risk for type 2 diabetes. Plant polyphenols such as diosmin, curcumin, and morin have the ability to constrain carbohydrate‐digesting enzymes. The extract of *Gardenia jasminoides* plays an important role in impeding α‐amylase and α‐glucosidase, thus reducing the fasting blood glucose level and high‐fat diet lipids. Epigallocatechin gallate (EGCG) and quercetin have the potential to reduce starch digestion by inhibition of α‐amylase activity (Li et al. 2025; Wu et al. 2024).

#### 3.4.4 Angiotensin‐Converting Enzyme (ACE) Inhibition

**FIGURE 8:**
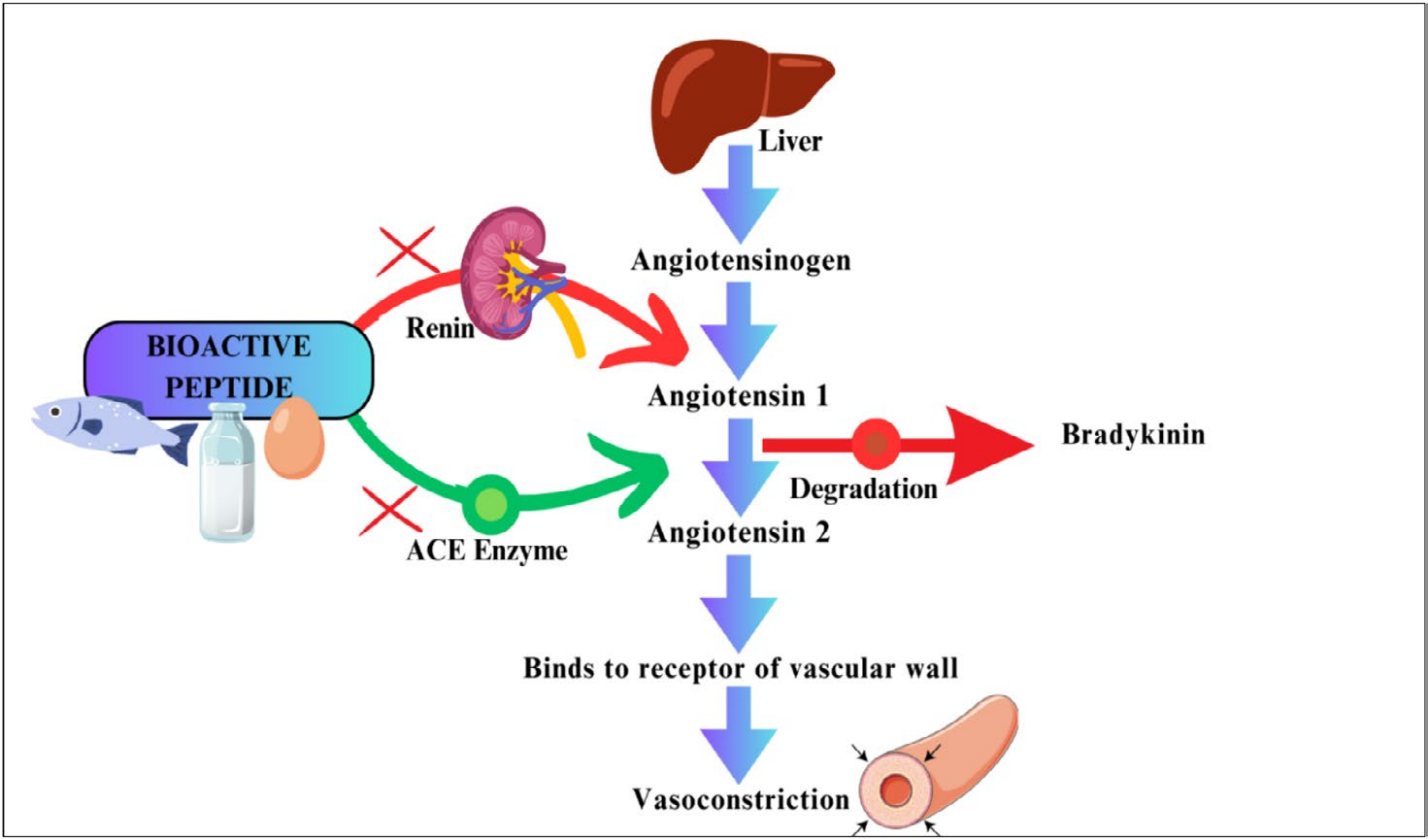
Anti‐hypertensive mechanism of bioactive peptides in functional food. https://www.canva.com/design/DAGjY‐O9P98/9cm2hhhkbeAAimd3Tzvs6A/edit?utm_content=DAGjY‐O9P98&utm_campaign=designshare&utm_medium=link2&utm_source=sharebutton.

Angiotensin‐converting enzyme converts angiotensin I to angiotensin II, a strong vasoconstrictor that is vital for the maintenance of blood pressure (Khurana and Goswami 2022). Peptides that are derived from fish proteins, fermented dairy, and soy emulate the action of pharmacological ACE inhibitors, thus inhibiting the active site of the enzyme and preventing the synthesis of angiotensin II synthesis (Figure 8). Flavonoids from green tea, cocoa, and grapes increase nitric oxide inhibition along with the inhibition of ACE, leading to improved endothelial function by vasodilation. Meta‐analytical evidence suggests that 3–5 g peptide/day may be effective in exerting antihypertensive effects without adverse outcomes (Ahmad et al. 2023; Hadi et al. 2022).

## 4 Applications in Functional Food Development

### 4.1 Fortified Beverages

Beverage is a liquid drink meant for human consumption in order to quench one's thirst, and it constitutes human culture. Fortified beverages, also known as designer beverages, are one of the major advancements in the development of functional food, and they provide increased nutritional advantages by incorporating different bioactive ingredients (Ajeeshkumar et al. 2021). These drinks are made to meet certain health needs, such as boosting probiotic viability, improving antioxidant activity, and supplementing essential nutrients. Functional ingredients, including probiotics, dietary fibers, omega‐3 fatty acids, and vitamins, are most commonly used in the fortification of beverages (Afzal et al. 2022).

#### 4.1.1 Examples and Applications

Emmer‐based fermented beverages enriched with fruit juices (e.g., blueberry, aronia) are non‐dairy probiotic alternatives, especially suitable for lactose‐intolerant individuals. These beverages, when fortified with *Lactiplantibacillus plantarum* and fruit juices like blueberry or aronia, deliver total phenolic contents and antioxidant capacities while maintaining probiotic viability above during refrigerated storage (Dimitrellou et al. 2021; Pannerchelvan et al. 2023). Fortified juices are the valuable alternatives that can be consumed by lactose‐intolerant individuals. Evolus from Valio Finland is a commercial beverage that is supplemented with bioactive peptides from *Lactobacillus helveticus* (Jauhiainen et al. 2005). Synbiotic beverages that are fortified with prebiotics and cereal sprouts demonstrate enhanced sensory acceptance and probiotic viability, catering to health‐conscious consumers (Kheto et al. 2025).

### 4.2 Functional Dairy Products

Functional dairy products have become an essential component of the food industry, which contributes to human health beyond mere nutrition. The adaptability of dairy ingredients, along with advancements in the dairy industry, has enabled the development of a diverse array of functional dairy products (Khalaf et al. 2021). These products can easily be incorporated with essential bioactive components, including bioactive peptides, prebiotics, probiotics, and other functional ingredients, that make a versatile dairy products in health‐promoting foods (Lamsar and Abhilasha 2023).

#### 4.2.1 Probiotics and Prebiotics Dairy Products

The dairy matrix offers a protective milieu for probiotic bacteria along with prebiotics, enhancing their survival during storage and processing (Ali et al. 2022). According to studies, yogurt is fortified with *Bifidobacterium bifidum, Lactobacillus acidophilus*, and *Streptococcus thermophiles* to balance the gut microbiome.

**Activia** by Danone's yogurt, which is a functional food, contains probiotic strain *Bifidobacterium animalis* DN‐173010 and insulin. Clinical studies report faster intestinal transit and symptom relief in individuals with IBS.

**Yakult**, a fermented dairy drink, delivers *Lacticaseibacillus paracasei* strain Shirota (LcS) to improve digestion (according to Yakult Philippines 2019). Studies show regular intake reduces constipation episodes, improves gut immunity, and is consumed in over 40 countries (Lamsar and Abhilasha 2023).

#### 4.2.2 Fortified Dairy Based Bioactive Peptides and Other Dairy Functional Products

The philosophy of science aims to enhance the quality of human life, and for many years, bioactive peptides have been used as a component of functional food to improve this quality. Bioactive peptides are biological entities that are buried inside precursor proteins and become functional during cleavage of that protein (Akbarian et al. 2022). Peptides derived from casein and whey have shown to inhibit ACE; therefore, reducing blood pressure.

**Calpis**, from Japan, contains Val‐Pro‐Pro (VPP) and Ile‐Pro‐Pro (IPP) tripeptides, which inhibit ACE activity and are clinically shown to reduce systolic blood pressure in mildly hypertensive individuals (Table 5).

**TABLE 5:** Some commercially available functional dairy products.

Brand/product name	Type of product	Active ingredient	Nutrient content/serving	Consumer outcomes	Manufacturer	References
Probiotics						
Yakult	Fermented milk	*Lactobacillus casei* Shirota	160 cal, 1 g protein, 14 g carbohydrates	Prevents constipation and gastrointestinal infections	Yakult Honsha Co., Japan	Mellentin (2023)
Biobalance	Fermented milk	*Bifidobacteriumlactis*	7 cal, 1 g of total carbohydrates	Facilitates intestinal transit	Dos Pinos R.L., Costa Rica	Ali et al. (2022)
Activia	Yogurt	*Bifidobacteriumanimalis* DN‐173, *Bifidusregularis*, *Bifidusactivo*	90 cal, 1.5 g of total fats, 1 g of saturated fats, 5 mg of cholesterol, 55 mg of sodium, 15 g of carbohydrates, 12 g of total sugars, 4 g of proteins	Controls irritable bowel syndrome	Groupe Danone, France	Ali et al. (2022)
Prebiotics						
Orafti	Soluble powder	Inulin/oligofructose	101 kcal, 1.5 g total fat, 1 g saturated fats, 5 mg cholesterol, 55 mg sodium, 20 g total carbohydrates, 12 g total sugars, 4 g proteins	Improves intestinal flora	BENEO Group, Belgium	Kumar et al. (2022)
Hilma	Fiber + enzyme supplement	Acacia gum, psyllium husk	20 cal, 6 g total carbohydrates; 6 g dietary fiber, 100 mg enzyme blend	Supports bowel regularity, reduces bloating, improves digestion for sensitive stomachs; prebiotic support for gut microbiome	Hilma, USA	Hilma (2020)
Bioactive peptides						
Recaldent	Ingredient	Caseinophosphopeptides and amorphous calcium phosphate	18.6 kcal per 8 pastilles (10.4 g)	Anti‐cariogenic activity	Cadbury Co. UK	Ali et al. (2022)
Calpis	Sour milk	Hypotensive tripeptides	~50 kcal, ~12 g carbohydrates/sugars, ~15 mg sodium, ~20 mg calcium	Reduces blood pressure	Calpis Co., Japan	Peighambardoust et al. (2021)
Animal‐Derived Functional Compounds from By‐products						
Collagen peptides (from fish)	Animal‐derived (waste‐based)	Marine collagen peptides	369 cals, 90 g of proteins	Enhances skin elasticity, supports joint health	Vital proteins (marine collagen)	Steele (2022)
Chitosan‐based drinks	Functional animal product	Chitosan (from shrimp/crab shells)	3 kcal, 0.5 g total carbohydrates, 0.5 g dietary fiber	Fat‐binding, cholesterol‐lowering, weight management, digestive health	Various nutraceutical companies	Ylitalo et al. (2002)
Eggshell membrane hydrolysates	Nutraceutical capsule/drink	Collagen, glycosaminoglycans	66 mg of collagen and 100 mg of hyaluronic acid	Joint support, reduces inflammation, improves connective tissue health	Multiple nutraceutical brands	Ruff et al. (2009)
BioPURE‐GMP	Nutraceutical capsule/drink	Glycomacropeptides (GMP)	100 cals, 16 g of proteins, 3 g of fats, and 2 g of carbohydrates, 75 mg of cholesterol	Enhances gut barrier function, exhibits anti‐carcinogenic properties	Davisco Foods International, USA	Rackerby et al. (2024)

Research also proved that consumption of **omega‐3 fortified milk** for approximately 12 weeks led to improved brain function in older individuals. In Canada and the US, milk is supplemented with vitamin D to prevent osteoporosis and rickets (Peighambardoust et al. 2021).

#### 4.2.3 Functional Animal‐Derived Products From Wastes

Bioactive compounds from animal waste streams and by‐products are being recycled into functional foods and nutraceuticals in response to concerns about sustainability and the circular economy. These include:

**Fish‐derived collagen peptides** (from skin, scales, and bones) have been added to drinks, jellies, and capsules to promote healthy joints and skin. Research shows that consistent consumption improves skin elasticity and lessens joint pain (Steele 2022).

**Chitosan** (from shrimp and crab shells) has been used in fat‐binding drinks, antimicrobial packaging, and dietary supplements that lower cholesterol and help in weight management (Ylitalo et al. 2002).

**Eggshell membrane hydrolysates**, enriched with collagen and glycosaminoglycans, are used in functional drinks and capsules to support connective tissue and joints (Ruff et al. 2009).

**Glycomacropeptides (GMP)** from cheese whey are present in products like **BioPURE‐GMP**. It shows anti‐carcinogenic properties and enhances gut barrier function (Rackerby et al. 2024).

These innovations demonstrate how animal‐derived by‐products are being transformed into high‐value health‐promoting ingredients, reducing waste and enhancing sustainability in the food sector.

### 4.3 Poultry as a Source of Functional Compounds

Poultry products (e.g., ducks, chickens, and turkeys), beyond providing basic nutrition, are a significant source of functional compounds that include antioxidants, bioactive peptides, and vital nutrients like omega‐3 fatty acids and amino acids (Romero‐Garay et al. 2022).

The cardiovascular benefits of **omega‐3‐enriched eggs** have recently drawn the attention of the researchers. It has been reported that hens supplemented with flaxseed or fish oil exhibited enhanced content of alpha‐linolenic acid (ALA), eicosapentaenoic acid (EPA), and docosahexaenoic acid (DHA) in their eggs. This provides a practical way to raise omega‐3 intake in the general population (Vlaicu et al. 2021).

Additionally, **carotenoids** like lutein and zeaxanthin, which are critical for eye health, can be delivered through poultry products. Adding carotenoid‐rich feed ingredients, such as algae or marigold extract, improves carotenoid content and yolk color of eggs (Dansou et al. 2023).

**Antioxidant capacity and vitamin enrichment** in poultry can be manipulated through dietary strategies. For example, the inclusion of plant‐derived polyphenols or vitamin E in poultry feed not only improves the oxidative stability of meat and eggs but also potentially enhances their health‐promoting properties for consumers (Matumoto‐Pintro et al. 2017).

### 4.4 Snack Foods

The increased demand for healthy, on‐the‐go food options has fueled innovation in formulations of functional snacks, thus integrating scientifically validated ingredients to boost nutritional profile (Krüger et al. 2022). Researchers formulated a snack bar which contained a blend of 25% oats, 15% banana peel powder, and 60% amaranth grain, involving all the essential components like phenolics, antioxidants, β‐glucan, and minerals. Thermogravimetric analysis revealed that its active components are stable at high temperatures, thus preserving functional capabilities. Sensory evaluations of indicated that this bar could be stored for about 60 days without being contaminated and also has both nutritional and economic benefits (Boukid et al. 2022).

Although using banana peels provides a nutrient‐rich and sustainable alternative, there are certain limitations associated with its use (Deb et al. 2022). Potential issues include the indigestibility of some fibrous components and the presence of heavy metals (such as lead and cadmium) from environmental exposure, which require careful regulatory review to guarantee food safety and consumer acceptance. These factors must be addressed for the commercialization of functional innovations (Yasin et al. 2025).

#### 4.4.1 Bioactive Components Containing Functional Food Snacks

Residues of *Hibiscus sabdariffa*, significantly containing flavonoids and phenolics, were incorporated into snack crackers to boost their antioxidant potential DPPH scavenging activity potential (Chew et al. 2024). For example, Dang coconut chips contain magnesium and selenium as minerals that activate glutathione peroxidase, which combats oxidative stress. Similarly, Sunroot (Jerusalem artichoke) is supplemented with snack foods due to its rich content of antioxidants and insulin (Nazzaro et al. 2025). *Cannabis sativa* contains a bioactive component, cannabinoids, as a functional component, and the inclusion of this plant seed in the dough of gluten‐free bread and crackers that is rich in iron contributes toward reducing disease risk (Krüger et al. 2022).

However, it's crucial to note that only hemp‐derived strains of *Cannabis sativa* with low optimized levels of tetrahydrocannabinol (THC) are utilized in culinary applications. There is no risk of psychosis or addiction because these hemp seeds don't contain large amounts of cannabinoids like CBD or THC (EFSA 2022; FDA 2024). Strict regulations govern the use of hemp‐derived food ingredients; for example, the European Food Safety Authority (EFSA) and the US Food and Drug Authority (FDA) permit the use of hemp seeds in food products as long as the THC content stays below predetermined limits (usually ≤ 0.3%) and no cannabinoid extraction is done (EFSA 2022; FDA 2024).

### 4.5 Dietary Supplements

**FIGURE 9:**
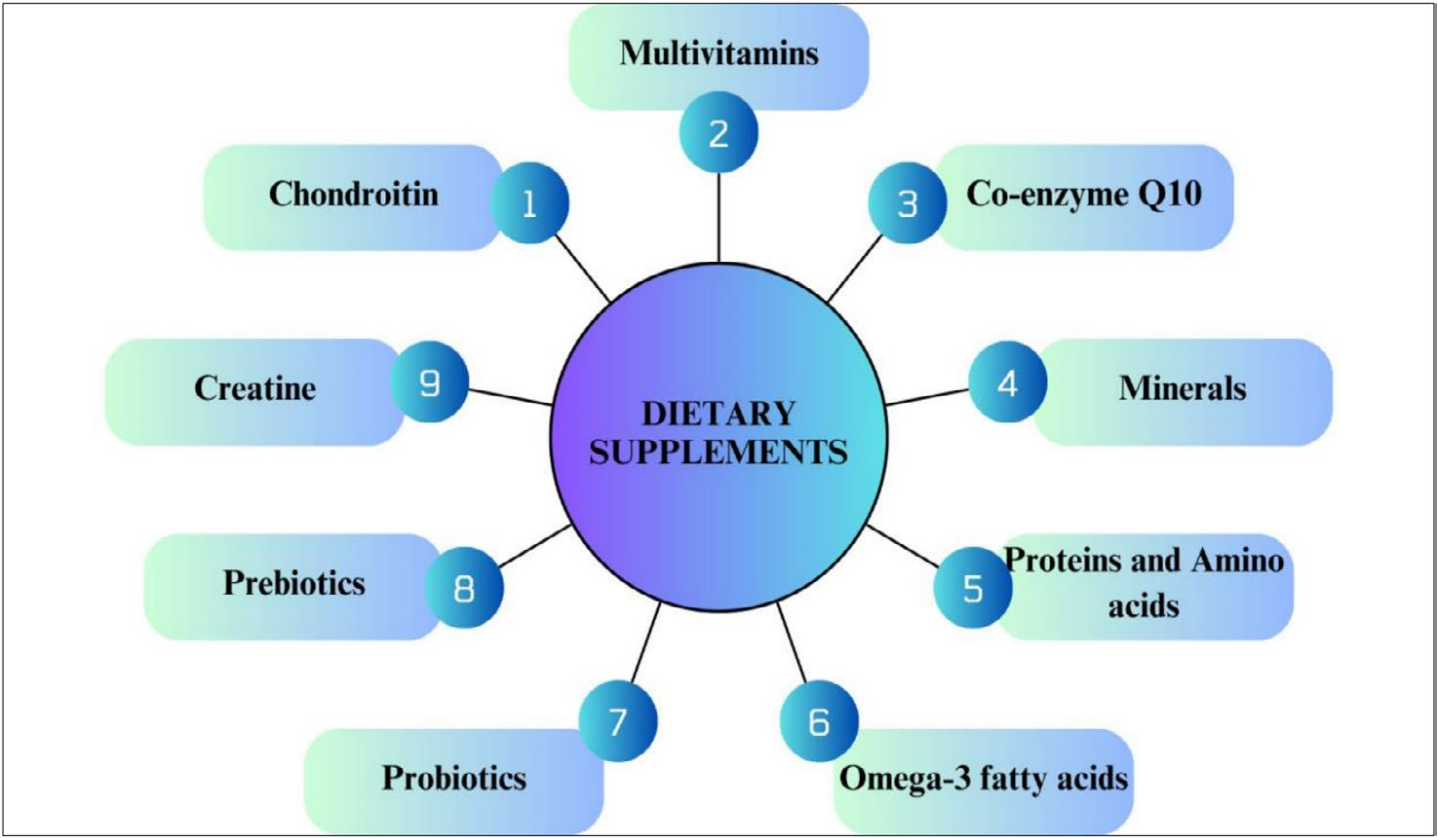
Functional food‐based dietary supplements. https://www.canva.com/design/DAGjZEHPiwM/moppQ8cF6fhMfJcCjyhp‐g/edit?utm_content=DAGjZEHPiwM&utm_campaign=designshare&utm_medium=link2&utm_source=sharebutton.

The global rise in chronic ailments, consumer demands for health‐promoting foods, and nutrient deficiencies has resulted in the expansion of dietary supplement‐enriched functional foods. According to the 1994 Dietary Supplement and Education Act, nutritional additives include vitamins, minerals, amino acids, proteins, fatty acids, and fiber, which are known as dietary supplements (Alongi and Anese 2021). Vitamin D dairy products have been demonstrated to improve immunological and bone health. Iron‐supplemented cereals have been developed to fight anemia, especially in populations where iron deficiency is highly prevalent. PUFA ꞷ‐3 and ꞷ‐6 and MUFA ꞷ‐9 in functional foods reduce the chance of inflammatory disease and coronary heart disease. Coenzyme Q‐10 is an internal lipophilic that is effective as a dietary supplement in functional food effective for human fitness and skin smoothness (Figure 9). Resveratrol is a polyphenol that is found in grapes, integrated into beverages for longevity‐promoting properties (Boggia et al. 2020).

**TABLE 6:** Global market value of functional foods.

Category	Market highlights	References
Global market shares		
Global market value (2021)	**USD 177.4B**, Projected **USD 219.5B by 2026**	Sørensen et al. (2022)
Pandemic impact	Growth rose from **0.3% (2019–20)** to **5% (2020–21)**, driven by health awareness (e.g., vitamin D fortification)	Sørensen et al. (2022)
Europe (2021)	Western Europe: **USD 23B**, Eastern Europe: **USD 5.2B**. UK (20% of EU), Germany (14%), France (13%), Spain (12%), Italy (11%)	Sørensen et al. (2022), Vicentini et al. (2016)
Latin America	Mexico: **USD 12.7B (2011)** → **USD 16.3B (2015)**, Rapid expansion: **Brazil +10% annually**	Sørensen et al. (2022), Vicentini et al. (2016)
Other regions	Saudi Arabia, Israel, and South Africa show higher functional food potential Eastern Europe has fragmented functional food market Within the region, Russia dominates the market, accounting for 51% of all functional food sales in Eastern Europe	Sørensen et al. (2022), Vicentini et al. (2016)

*Note:* Bold values highlight key market sizes, growth rates, and dominant regional contributions.

**TABLE 7:** Types of beneficial functional foods available in the market with brand names.

Product/functional food	Type	Key bioactive compound(s)	Company/brand	Regional availability	Retail price range (USD)	Reported health benefits	References
Activia (probiotic yogurt)	Dairy/yogurt	*Bifidobacteriumanimalis* subsp. *Lactis*	Danone (Dannon)	USA, EU, APAC	$1.0–$2.5 (single cup)	Supports digestive health; reduces minor GI discomfort	Danone (2023)
Yakult (probiotic drink)	Fermented beverage	*Lactobacillus casei* Shirota	Yakult Honsha	Global (major markets)	$0.8–$1.8 (single bottle)	Supports gut microbiota and regularity	Kato‐Kataoka et al. (2016)
DanActive/Actimel	Fermented beverage	*L. casei* strains + vitamins D/B6	Danone	USA, EU, APAC	$1.0–$2.0 (single bottle)	Immune support; digestive health	Danone (2023)
Special K (fortified cereal)	Cereals	Added vitamins & minerals (iron, B vitamins)	Kellogg's	Global	$3–$6 (box)	General micronutrient fortification	kelloggs.co.uk, n.d.
Eggland's Best (Omega3 EGGS)	Eggs	Omega3 fatty acids (ALA, EPA/DHA)	Eggland's Best	USA, EU markets	$3.5–$6.5 (dozen)	Increased omega3 intake for CV health	Michella and Slaugh (2000)
GT's SYNERGY Kombucha	Kombucha	Live cultures, tea polyphenols	GT's Living Foods	USA, EU, APAC (selected)	$3–$5 (bottle)	Gut health, antioxidants	GT's Living Foods
Beyond Burger (plant based patty)	Plant based meat	Pea/rice proteins; fortified iron	Beyond Meat	Global (major retailers)	$4–$8 (pack)	Protein alternative with lower sat fat	Beyond Meat (2024)
Quest protein bars	Snack bar	High protein (whey/milk isolates), fiber	Quest Nutrition	USA, EU	$1.8–$3.5 (bar)	Muscle maintenance, satiety	Quest Nutrition (2024)
Manitoba harvest hemp hearts	Seeds/toppings	‐Omega3 & omega6 fatty acids, plant protein	Manitoba Harvest	North America, EU (selected)	$6–$12 (8 oz)	Plant protein & essential fatty acids	Manitoba Harvest Hemp Foods (2025)
Huel (meal replacement)	‐Meal replacement Powder/ready to drink	Balanced macros +27 essential vitamins & minerals	Huel Ltd.	Global (online + retailers)	$2.0–$4.5 per meal equivalent	Complete nutrition/weight management	HuelLimited (2015–2025)
Ensure (clinical nutrition)	Nutrition shake	Proteins, vitamins & minerals (27+), ALA in some formulations	Abbott Nutrition	Global	$1.5–$3.5 (bottle)	Clinical nutritional support; elderly nutrition	Abbott (2025)
Oatly (fortified oat milk)	Plant based milk	Added calcium, vitamin D, B12	Oatly	Global (major markets)	$2.5–$5 (carton)	Dairy alternative with fortification	oatly.com, n.d.
Chobani (probiotic yogurt lines)	Dairy/yogurt	Live cultures (probiotics)	Chobani	USA, select international	$1.0–$2.5 (cup)	Digestive health; protein‐rich yogurt	Chobani (2024)
Alpro (fortified plant drinks)	Plant based beverage	Vitamins & minerals (Ca, B12, D)	Alpro (Danone)	EU, UK, APAC	$2–$4 (carton)	Dairy alternative with micronutrients	Alpro (2025)
Nature's Bounty Omega3 (supplement)	Dietary supplement	EPA/DHA fish oil	Nature's Bounty	Global	$8–$20 (bottle)	Cardiovascular & cognitive support	Bounty (2025)
Optimum Nutrition Gold Standard Whey (Protein)	Sports nutrition	Whey protein isolate/concentrate	Optimum Nutrition (Glanbia)	Global	$1.0–$2.5 per serving	Muscle recovery and performance	Optimum Nutrition (2025)
S‐26 Probiotic Infant Formula	Infant nutrition	Probiotic strains (varies by formulation)	Wyeth/Nestlé/Mead Johnson variations	Selected regions	$20–$60 (tin)	Gut health in infants (formulation‐specific claims)	Nestlé (2025)
Olly (multivitamin gummies)	Dietary supplement/gummies	Vitamins (multi blends)	Olly	USA, online global	$8–$18 (bottle)	General wellness, sleep/beauty formulations	Unilever (2025)

## 5 Market of Different Types of Functional Foods

The global functional food market has emerged as one of the fastest‐growing sectors in the food industry, driven by consumer demand for preventive health solutions, rising healthcare costs, and increasing awareness of diet‐related diseases. According to Euromonitor, the global market for functional foods is estimated to be worth USD 177.4 billion in 2021 and expected to be USD 219.5 billion by 2026 (Sørensen et al. 2022; Tables 6 and 7). This discrepancy reflects differences in the classification of functional foods rather than an actual decline in market size.

## 6 Challenges Associated With Functional Foods

With compromised living standards and life expectancy, along with a high frequency of illnesses, nutrition plays an important role in maintaining human health. Scientists are working in collaboration to meet various food demands with associated therapeutic advantages, popularly known as functional food, promising in ensuring human well‐being and lowering the risk of illnesses (Lobine et al. 2022). The utilization of bioactive components and natural products in functional food development is a quickly expanding research area, providing significant health benefits beyond basic nutrition. However, multiple challenges must be addressed to secure their safety, efficacy, and commercial viability.

### 6.1 Bioavailability and Stability Issues

Bioavailability and stability are essential components in the development of functional foods, influencing their efficacy and shelf life (Mihociu et al. 2024).

Functional bioactive compounds like polyphenols, probiotics, peptides, and omega‐3 fatty acids often exhibit weak solubility, low permeability, and susceptibility to enzymatic destruction. Molecular size, hydrophobicity, and interaction with food matrices can also impede absorption (Mafe et al. 2025; Mihociu et al. 2024). Functional ingredients are susceptible to deterioration due to environmental variables such as temperature, pH, light, and oxygen. Oxidation, hydrolysis, and microbial contamination can all impair their structural integrity and efficacy. Stability is also a major concern during processing, storage, and digestion, requiring advanced delivery systems (e.g., nanoencapsulation, liposomes) (Mafe et al. 2025; Mihociu et al. 2024).

### 6.2 Standardization and Quality Control

Standardization and quality control are difficult for the formation of functional foods because of the complexity of bioactive substances, the unpredictability of raw materials, and inconsistent regulations. Exact analytical methods such as mass spectrometry and HPLC are necessary to guarantee consistent constituent composition; however, consistency is hampered by natural variances. Regional differences in regulations lead to discrepancies in safety evaluations, health claims, and labeling (Wani et al. 2023).

### 6.3 Regulatory and Safety Concerns

Functional foods are at the nexus of food and medicines, which presents challenges for their development due to complicated regulatory frameworks and safety issues. Regulatory agencies like the FDA and EFSA enforce strict rules on labeling, ingredient approval, and health claims; yet, regional variations make it difficult for products to be accepted on the international market. It takes sophisticated analytical techniques, such as mass spectrometry and microbiological testing, to guarantee the absence of pollutants, allergies, and poisons. It is also necessary to assess the long‐term consequences of intake through toxicological and clinical research (Vilas‐Boas et al. 2021).

### 6.4 Consumer Acceptances

Consumer acceptability and marketability, which are impacted by sensory qualities, perceived health advantages, and cost, are critical to the success of functional foods. Customers frequently reject functional food because of unfavorable flavor, texture, or appearance brought on by the addition of bioactive ingredients. Acceptance is further hampered by false information and mistrust over health claims, which makes open communication and claims supported by science necessary. Furthermore, market penetration is restricted by premium pricing brought on by expensive R&D and sophisticated processing methods (Grinberga‐Zalite et al. 2024).

### 6.5 Cost–Benefit Analysis of Functional Food Versus Regular Food

In addition to regulatory and safety concerns, economic feasibility remains a critical challenge influencing the widespread adoption of functional foods as they may carry a higher price point due to added bioactive components and enhanced processing. A comparative cost–benefit analysis indicates that preventive intake of functional foods could outweigh the initial cost when weighed against medical expenses for managing lifestyle‐related disorders; however, several studies suggest their long‐term cost‐effectiveness in reducing healthcare burdens associated with chronic diseases (Mehboob 2025; Michaud et al. 2017; Musich et al. 2016). Nonetheless, affordability and equitable access remain key barriers to consumer adoption, especially in low‐ to middle‐income populations (Zheng 2022).

### 6.6 Advancement in Biotechnology and Omics Approaches

**FIGURE 10:**
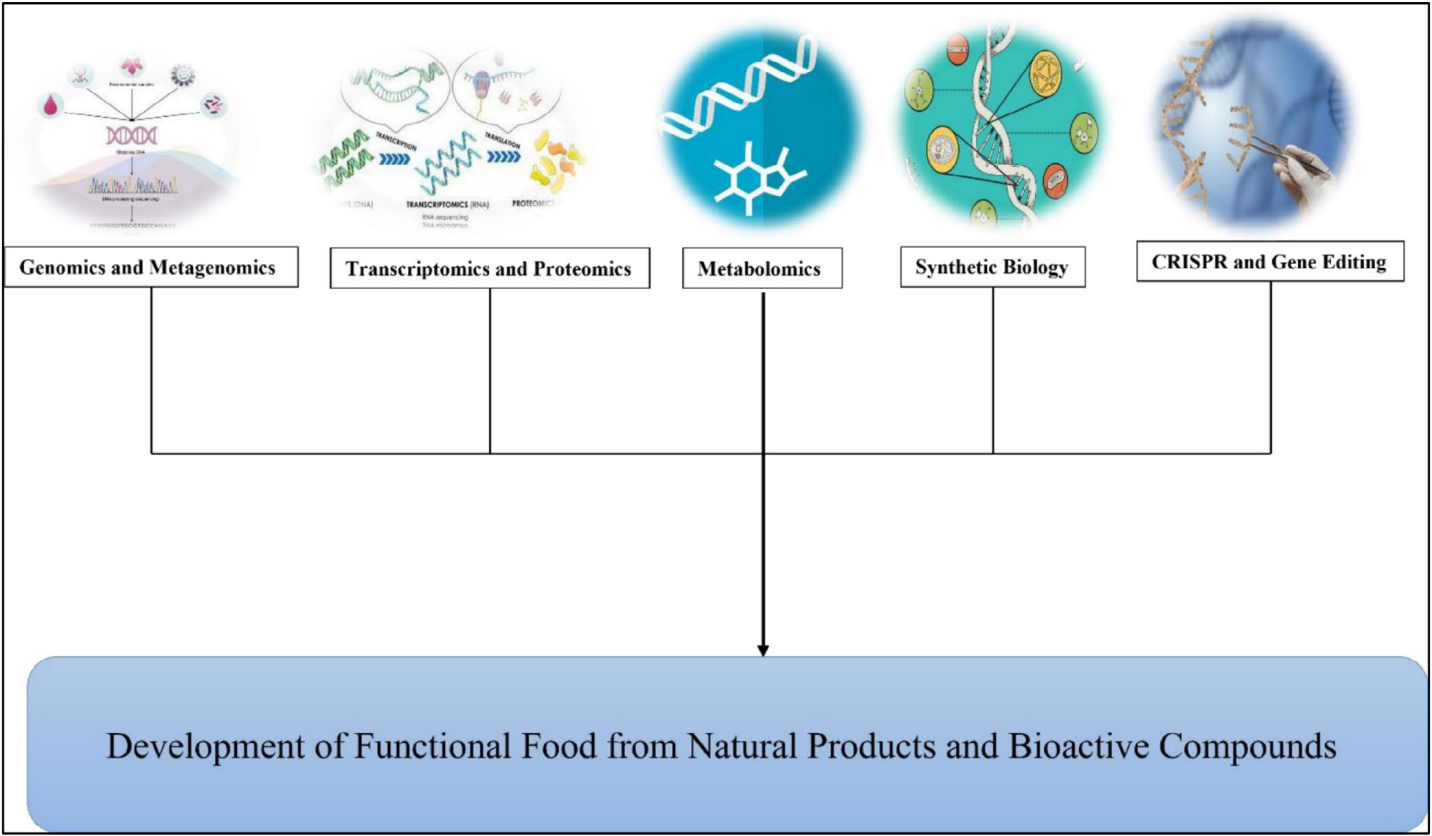
Advancements in biotechnological and omics approaches.

The “omics technologies and biotechnology” have revolutionized the discovery, characterization, precision, and application of bioactive compounds and natural products in the functional food development. Various approaches are being used, including “omics”, synthetic biology, genetic engineering, and gene‐editing (Figure 10). These approaches provide a deeper knowledge of complex interactions between human biology and bioactive molecules, opening ways to ward targeted, validated, and effective functional foods (Nayak et al. 2021).

### 6.7 Development of Smart Delivery Systems

Smart delivery system in functional food development enhancing the bioavailability, stability, and targeted release of sensitive bioactive compounds. Techniques such as liposomes, biopolymer‐based carriers, and nano‐emulsions protect bioactive compounds and improve their absorption. Additionally, smart packaging with intelligent and active features helps maintain product quality and increase shelf life, ensuring efficacy from production to consumption (Shishir et al. 2021).

### 6.8 Personalized Nutrition and Functional Foods

Personalized nutrition in the development of functional food offers tailored dietary solutions based on an individual's metabolism, lifestyle, and genetics. Researchers can develop customized functional food products by using tools such as nutrigenomics, microbiome analysis, and metabolomics that effectively target specific health conditions. Advancements in digital health and AI technologies further enable personalized recommendations and real‐time monitoring, enhancing the effectiveness, accessibility, and precision of functional nutrition (Dj Nevena et al. 2022).

### 6.9 Expanded Clinical Research and Regulatory Harmonization

Expanded clinical research and regulatory harmonization are crucial for advancing functional foods derived from natural products and bioactives. Strict clinical trials are essential to establish the safety, efficacy, and health benefits of these foods, paving the way for personalized nutrition and improved consumer acceptance. Meanwhile, regulatory harmonization creates global standards that ensure product quality and safety, facilitating innovation and international trade. By promoting collaboration among regulatory bodies, researchers, and industry stakeholders, these efforts will increase the development and trust in functional foods, ultimately meeting the growing consumer demand for health‐promoting products (Alongi and Anese 2021).

## 7 Future Prospect and Conclusion

Bioactive compounds obtained from natural food products and plant extracts possess incredible supplementary and medicinal properties. These biologically active components such as carotenoids, probiotics, prebiotics, and omega‐3 fatty acids have diverse health benefits such as anti‐inflammatory, antioxidant, enzyme inhibition activities, and gut microbiome modulation. Modern biotechnological and bioinformatics approaches have revolutionized the development, characterization, and targeted delivery of these therapeutically and nutritionally significant natural compounds.

In the future, integrating precision nutrition strategies (“omics” technologies and machine learning) may accelerate the discovery of new bioactive compounds and customized therapeutics. Sustainable sourcing, enhanced bioavailability through nanotechnology, and thorough clinical validation of these compounds will ensure their effectiveness, safety, and widespread adoption. To bridge the gap between conventional approaches and contemporary healthcare innovations, these natural compounds must also be transformed from research to mainstream health solutions in the market.

## Author Contributions

A.H. and Z.A.: Conceived and designed the project; Z.A., S.S., A.H., E.Y., and M.R.: analyzed, wrote, revised, and proofread the manuscript. All authors contributed to the article and read and approved the final manuscript.

## Ethics Statement

The authors have nothing to report.

## Consent

The authors have nothing to report.

## Conflicts of Interest

The authors declare no conflicts of interest.

## Data Availability

Data used during the preparation of this manuscript is available within the article.
